# Transfer Calibration Validation Tests on a Heat Flux Sensor in the 51 mm High-Temperature Blackbody

**DOI:** 10.6028/jres.106.039

**Published:** 2001-10-01

**Authors:** A. V. Murthy, B. K. Tsai, R. D. Saunders

**Affiliations:** Aero-Tech, Inc., Hampton, VA 23666; National Institute of Standards and Technology, Gaithersburg, MD 20899-8441; Saunders Enterprises, Gaithersburg, MD 20878

**Keywords:** blackbodies, heat flux, sensors, thermal radiation, transfer calibration

## Abstract

Facilities and techniques to characterize heat flux sensors are under development at the National Institute of Standards and Technology. As a part of this effort, a large aperture high-temperature blackbody was commissioned recently. The graphite tube blackbody, heated electrically, has a cavity diameter of 51 mm and can operate up to a maximum temperature of 2773 K. A closed-loop cooling system using a water-to-water heat exchanger cools electrodes and the outer reflecting shield. This paper describes the newly developed blackbody facility and the validation tests conducted using a reference standard Schmidt-Boelter heat flux sensor. The transfer calibration results obtained on the Schmidt-Boelter sensor agreed with the previous data within the experimental uncertainty limits.

## 1. Introduction

Development of blackbody facilities and techniques to calibrate high heat flux sensors has been in progress at the National Institute of Standards and Technology (NIST). Reference [1] gives an overview of the present status of the various calibration methods in use and the facilities under development. Presently, calibrations of heat flux sensors are performed in a 25 mm diameter cavity graphite tube Variable-Temperature Blackbody (VTBB) facility. The calibration method, known as the transfer technique [2], uses an electrical substitution radiometer (ESR) as a transfer standard and the VTBB as the transfer source. The transfer technique has been extensively used in the last four years to calibrate various sensors up to heat flux levels of approximately 50 kW/m^2^. An in-house reference standard Schmidt-Boelter sensor was also calibrated frequently during this period to study the long-term repeatability of the calibration technique. Several calibrations on this reference sensor have shown that the long-term repeatability over this period is within the expanded measurement uncertainty limits of ±2 % with a coverage factor^1^, *k* = 2.

To perform calibrations at heat flux levels higher than 50 kW/m^2^, a new VTBB^2^,^3^ with a larger cavity diameter of 51 mm was recently commissioned. The large cavity diameter also facilitates development of a closed mode calibration technique, which involves placing the test sensor inside the blackbody cavity. This paper gives details of the new 51 mm VTBB facility and the results of calibrating the reference standard Schmidt-Boelter sensor using the transfer technique. The results are compared with earlier test data obtained with the 25 mm VTBB.

## 2. Facility Description

**Fig. 1:**
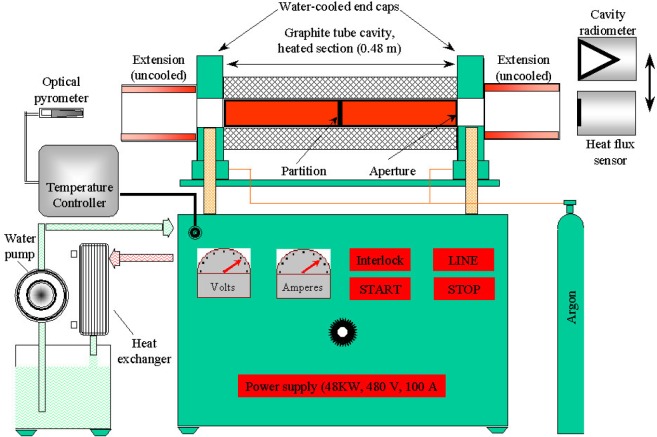
Schematic layout of the 51 mm variable temperature blackbody facility (VTBB).

Figure 1 shows a schematic layout of the 51 mm VTBB facility. The facility is composed of three major sections: the furnace, the power supply, and the cooling system. The furnace or blackbody cavity is a cylindrical tube made of graphite. Furnace heating is by direct resistance of the graphite tube using large ac currents at low voltages.

The heated section of the tube has a cavity diameter of 51 mm and is 0.48 m long with a 3 mm thick center partition. The tube is supported between two water-cooled copper end-caps, which in turn are connected to the electrode posts. The cooled copper end-cap, electrodes, and the heated tube are designed to provide a uniform temperature distribution along the cavity. The dual cavity design is useful in heat flux sensor calibration. The heat flux sensor to be calibrated and the reference radiometer are mounted on one side of the cavity. An optical pyrometer mounted on other side of the cavity senses the radiation from the cavity. The temperature measured by the optical pyrometer is used in controlling the furnace temperature.

The 48 kW furnace power supply which heats the blackbody operates on AC line voltage of 480 V rated for 100 A. The furnace can be operated in the temperature range from 873 K to 2773 K. The maximum temperature is reached in about 800 s. The cavity is purged with argon gas during operation to minimize sublimation at high temperatures and oxidation at low temperatures. The argon gas flow rate during normal operation is about 0.24×10^−3^ m^3^/s. Larger flow rates are used before starting to blow out trapped air from cavity insulation. Two graphite extension tubes, not cooled, are fitted to the cooled copper end caps to provide a smooth flow passage for the argon gas.

The furnace can be operated in a manual or an external mode by selection from a front panel switch. The manual mode is generally used during start-up to warm up the furnace to the minimum operating point. In the external mode, a high precision microprocessor-based controller controls the furnace temperature. The temperature measured by the precision optical pyrometer (Fig. 1) is used to control the temperature of the blackbody furnace within ±0.1 K of the set value. The initial operation has demonstrated long-term stability of the blackbody temperature within these limits. The optical pyrometer is fitted with different apertures depending on the operating temperature. The controller set point temperature can be varied in steps of 0.1 K, 1 K, 10 K, and 100 K. The controller can also be interfaced to a computer using a standard RS-232 port.

The blackbody cooling system is a closed-loop system consisting of a water-to-water heat exchanger, a circulating pump and storage and expansion tanks for water. The circulating system provides a flow rate of about 0.82 L/s at 689 kPa pressure. The system has several safety-interlock features built-in to ensure adequate cooling at the highest temperature of operation. The blackbody furnace shuts down if the circulating water temperature exceeds 90 °C, if the flow rate falls below 0.6 L/s, when the chilled water supply to the cooling system falls below 0.3 L/s, or in the event of a power system failure. These safety measures are designed to prevent major damage to the heater element in the event of an emergency.

## 3. Experiments

Before the actual start of experiments with the heat flux sensor, the blackbody system was operated up to the maximum temperature to check temperature stability and control. After some initial trials, the system operated satisfactorily, with long-term temperature stability within 0.1 K, enabling sequential testing of heat flux sensors and the reference radiometer.

### 3.1 Radiometer and heat flux sensor

**Fig. 2:**
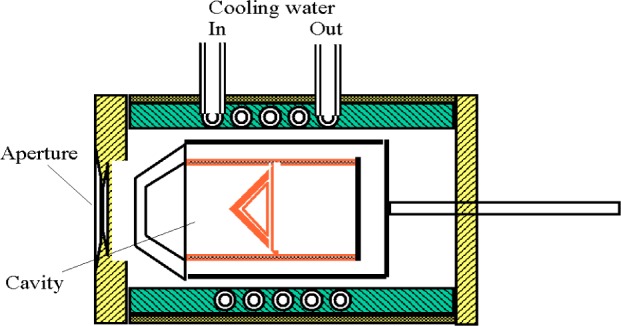
Schematic layout of the transfer standard radiometer.

The transfer technique validation experiments involved the calibration of a Schmidt-Boelter type heat flux sensor with reference to a transfer standard electrical substitution radiometer (ESR). The radiometer (Fig. 2) is water-cooled and operates at room temperature. The incident photon flux is nearly completely absorbed to within a fraction of a percent by multiple internal reflections within a black cavity. The electrical power required to produce the same temperature rise in the cavity as the incident flux is determined by measuring the voltage and the current through a precision resistor.

The radiometer used in the present calibration is rated for 4.2 W, and is fitted with a precision aperture (area = 1.0 cm^2^). The time constant (1/*e*) is about 6 s for a step change in irradiance. For large changes in heat flux level, the measurements are performed after about 60 s to allow for stabilization of the cavity temperature. The manufacturer’s stated accuracy of this radiometer is 0.5 % of the true value over the range of the instrument, as determined by an experimental determination of the Stefan-Boltzmann constant.

**Fig. 3:**
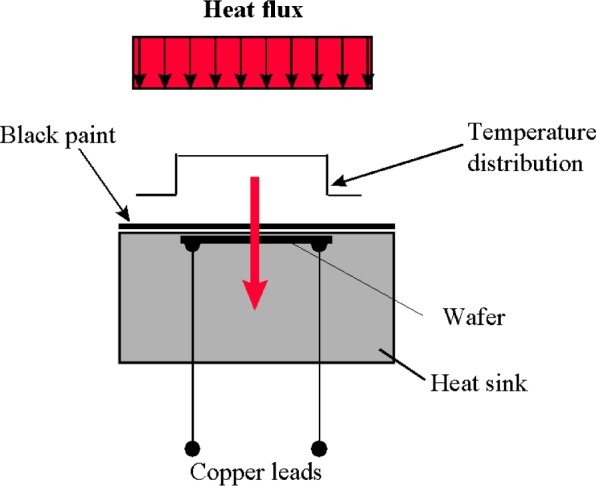
Schematic representation of a Schmidt-Boelter heat flux sensor.

The heat flux sensor used in the calibration is Schmidt-Boelter type. The Schmidt-Boelter sensor [3], shown schematically in Fig. 3, works on the principle of axial one-dimensional heat flow. It measures the temperature difference across a thin, thermally insulating layer to determine the incident heat flux. Due to the axial flow of heat, the temperature distribution across the sensing surface is uniform. The gage is 5 mm in diameter and 9 mm long, with a designed maximum heat flux of 110 kW/m^2^. This sensor has been calibrated with reference to the radiometer several times in the 25 mm VTBB facility up to 50 kW/m^2^ incident heat flux. These calibrations were repeatable within ±0.6 % with a measurement uncertainty of ±2 % (*k* = 2).

### 3.2 Test method

**Fig. 4:**
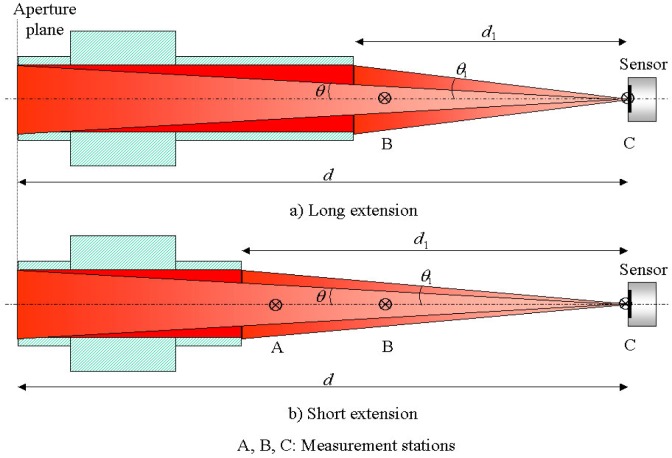
Location of sensor with reference to blackbody exit.

The blackbody has two graphite tube extension pieces, 25.4 cm and 17.4 cm, which fit on to the cooled copper end caps. The inside diameter of the tubes is the same as the blackbody cavity diameter. The short tube is used when calibrating at high heat flux levels so that the sensor can be located closer to the radiating aperture. Figure 4 and Table 1 give the geometric details of the sensor location with respect to the blackbody aperture and the extension pieces.

Tests were conducted with both the short and long extensions. The sensor and the reference radiometer were located at stations A, B, and C with the short extension piece installed on the blackbody. For the longer extension piece, the corresponding stations were only at locations B and C. After the blackbody temperature was stabilized at the set value, the radiometer and the heat flux sensor were placed in front of the black-body exit at stations A, B and C. Measurements were recorded for a period of 20 s to 100 s at approximately 0.4 s intervals using a digital multi-meter. The readings were averaged over the test time to account for the variations introduced due to argon purge gas effects. The purge gas effects were large when the instrument was close to the blackbody exit but diminished rapidly when it was moved away from the exit.

## 4. Results and Discussion

**Fig. 5:**
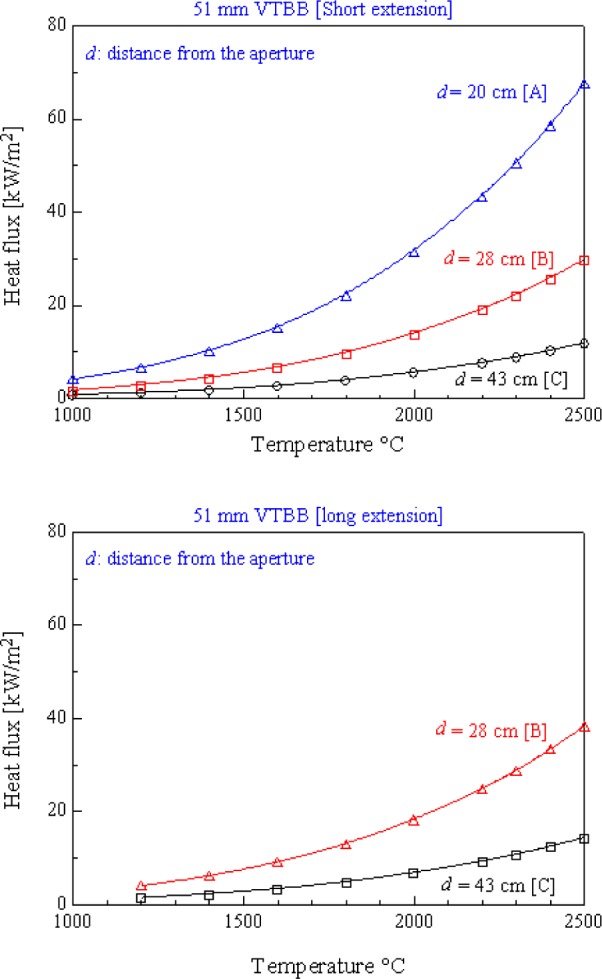
Measured heat flux levels at different distances from the blackbody exit [stations A, B, and C].

Figures 5 shows the measured heat flux levels at different distances *d* from the blackbody aperture, with increasing blackbody temperature. The location of the aperture is assumed to be at the end of the heating region of the graphite element. The distance d is used only as a reference to locate the sensor and the radiometer at a specified location.

The maximum heat flux obtained at station C, at a distance of d = 43 cm from the aperture with the long extension, is about 15 kW/m^2^ at the highest blackbody temperature of 2773 K. The corresponding maximum heat flux at station B (*d* = 28 cm) at the same temperature is about 40 kW/m^2^. With the short extension, the maximum measured heat flux at 2773 K is about 67 kW/m^2^, 30 kW/m^2^, and 12 kW/m^2^ at stations A, B, and C, respectively.

**Fig. 6:**
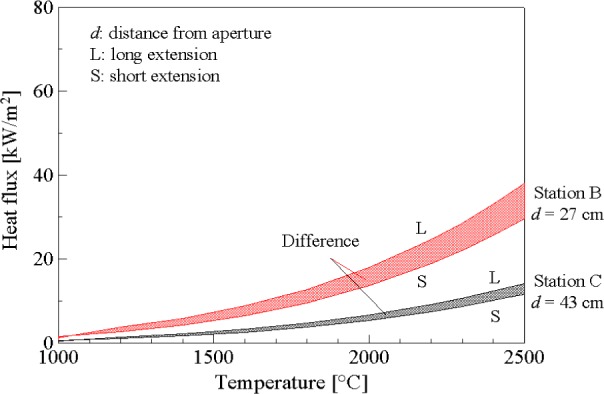
Reflected radiation from the graphite tube inner surface.

There is no view-limiting aperture mounted on the sensor or the radiometer. Hence, the heat flux measured by the sensor contains contributions from the direct radiation from the blackbody aperture and the radiation reflected from the inner surface of the graphite tube extension. The proportion of reflected radiation is higher for the longer extension tube compared to shorter tube. The shaded area in Fig. 6 shows the difference in reflected radiation at stations B and C between the two graphite tube extensions. The component of reflected radiation becomes smaller as the distance from the aperture increases.

Much of the radiation arriving at the gage surface is from a narrow view angle subtended by the blackbody aperture at the sensor. The reflected radiation, a smaller component of the total radiation, is at slightly larger angles. When the radiation incident at the sensor is at larger angles, a correction to account for the view angle is necessary. The value of the correction factor needs to be measured experimentally, particularly when the sensing surface of the gage is fitted with a view restrictor, or placed inside a cavity as in an ellipsoidal radiometer. When the sensor size is small compared to the radiating source and with no view restrictor, the correction factor is equal to the cosine of the angle between the normal to the sensor and the line joining the centers of the source and the sensor.

**Fig. 7:**
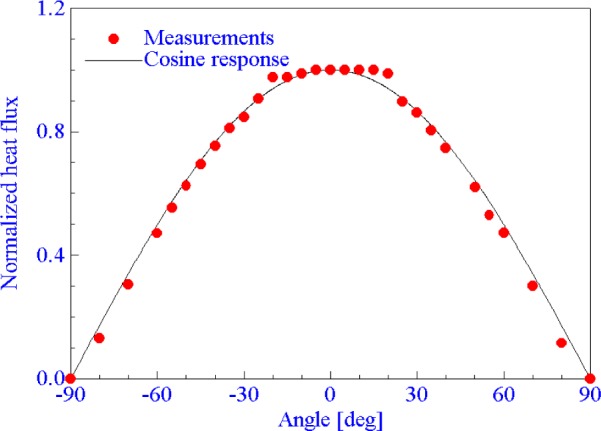
Angular response of the Schmidt-Boelter sensor measured in the 25 mm VTBB.

The cosine response of the heat flux sensor used in the present calibration was measured in the 25 mm VTBB. The measurements were made for different orientations of the sensor with respect to the radiant source operating at a fixed temperature of 2673 K giving a normal incident flux of 7 kW/m^2^ at the sensor surface. Measurement results along with the theoretical cosine response curve are presented in Fig. 7. The sensor output measurements for different angles have been normalized with respect to the value at the normal incidence (0°). Good agreement between the measurements and the theoretical response is observed, as expected.

**Fig. 8:**
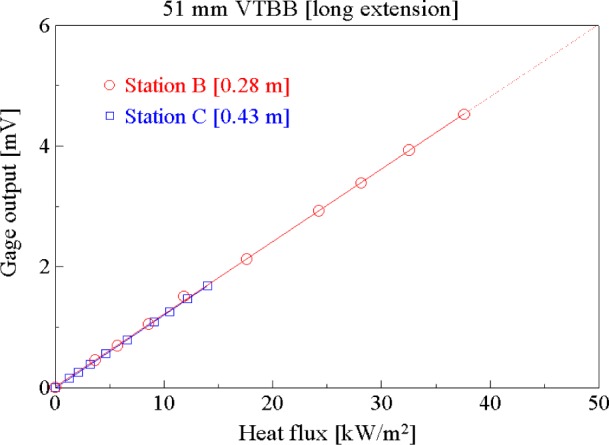
Schmidt-Boelter gage calibration in the 51 mm VTBB fitted with short extension.

**Fig. 9:**
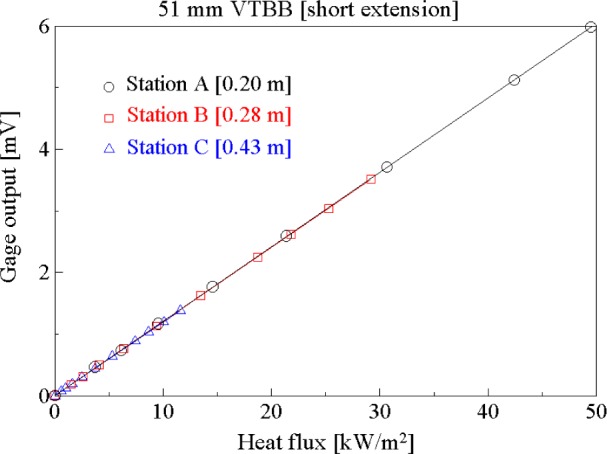
Schmidt-Boelter gage calibration in the 51 mm VTBB fitted with short extension.

Figure 8 presents the transfer calibration results obtained in the 51 mm VTBB with the standard long extension installed. The calibration results at stations B and C, at distances 0.28 m and 0.43 m from the aperture, agree closely indicating no significant effect of purge gas or other extraneous factors on the calibration. The corresponding calibration results for the case when the blackbody was fitted with a short extension are shown in Fig. 9. The results agree closely for all the three sets of measurements performed at stations A, B, and C with distances 0.20 m, 0.28 m, and 0.43 m, respectively, from the aperture. From this calibration data, the responsivity of the Schmidt-Boelter sensor was determined using linear regression. The regression factors for all the five sets of data, both with long and short extensions, were close to unity indicating good linearity of the sensor in the calibration range. The five sets of measurements, shown in Table 2, correspond to varying heat transfer, distance and tube extension. The agreement of the measured responsivity under these varying conditions supports the validity of the transfer technique in the 51 mm VTBB.

**Fig. 10:**
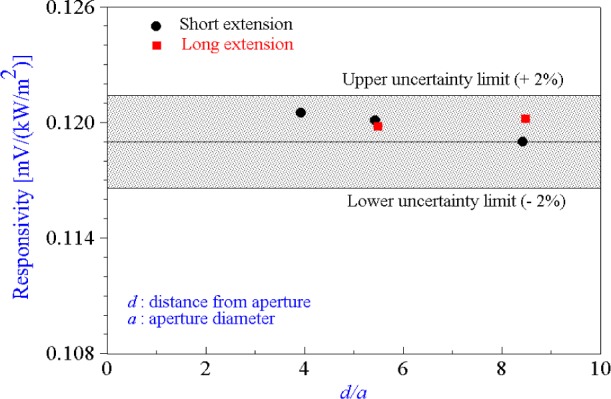
Comparison of Schmidt-Boelter calibrations in the 51 mm and 25 mm variable temperature blackbodies. Shaded area between solid lines represents the upper and lower uncertainty limits of the calibration in the 25 mm VTBB.

The responsivity also agrees within 1 % with the previous long-term repeatability data of the sensor in the 25 mm VTBB calibrations. This variation is well within the experimental relative expanded uncertainty of 2 % (k = 2). Figure 10 compares the present responsivity data with the previous calibrations in the 25 mm VTBB. It was observed with the 51 mm VTBB tests that the variance in the data due to purge gas effects was somewhat higher than observed during the test with the 25 mm VTBB. However, this effect does not influence the averaged data because of similar effects on both the radiometer and the sensor resulting in good agreement between the 25 mm and the 51 mm VTBB data. The purge gas flow rate in the 51 mm VTBB is about 0.24×10^−3^ m^3^/s, as compared to 0.08×10^−3^ m^3^/s for the 25 mm VTBB, because of the larger flow area. Reducing the purge gas flow rate to about 0.15×10^−3^ m^3^/s might decrease the variance in the sensor output data. However, this can lead to reduced life of the blackbody graphite heating-element.

## 5. Measurement Uncertainties

The measurement uncertainties are grouped under two categories: Type-A, evaluated using statistical methods, and Type-B, evaluated by other means such as previous measurement data, experience, manufacturer’s specification, and other sources of data [4]. The individual uncertainties are then combined using the square root of the sum-of-the squares to arrive at the combined standard measurement uncertainty. In the transfer technique calibration of heat flux sensors, the uncertainty accrues mainly from the measurement uncertainty at the two different stages: the transfer standard radiometer calibration uncertainty and the uncertainties at the various stages of calibration using the VTBB. The individual uncertainties are discussed below and the values tabulated in Table 3.

- Transfer standard uncertainty: The uncertainty associated with the calibration of the VTBB transfer standard is obtained from previous measurements. This is considered as a Type-B uncertainty and estimated to be 0.6 % [6].
- Blackbody radiation: The absolute temperature of the blackbody and the aperture emissivity has no influence on the transfer calibration because the heat flux is determined independently from the radiometer measurements. It is only necessary that the blackbody temperature is stable over the measurement time interval. The temperature stability is within ±0.1 K of the set temperature. The corresponding uncertainty in the radiant heat flux will be 0.04 % at 1000 K and 0.014 % at 2773 K, which is small compared to the other test uncertainties. The uniformity of radiation from the blackbody aperture has no major influence on the calibration because of identical effects on both the transfer standard and the test sensor.
- Alignment error: The test sensor and the radiometer are positioned using a gage block at a fixed distance from the blackbody exit, which is the reference plane. The effective radiating aperture is assumed to be at the end of the graphite heating element. Assuming a maximum error of about 0.2 mm in the location of the radiometer and the sensor with respect to the reference plane, the corresponding uncertainties in irradiance will be 0.2 %, 0.14 % and 0.09 % at sensor locations of corresponding to stations A, B and C. The errors due to angular misalignment vary as the cosine of the angle. Assuming a maximum misalignment of about 2°, the corresponding uncertainty will be 0.06 %.
- Sensor/Radiometer aperture area: Downstream of the radiating aperture, the heat flux distribution has a peak at the center and decreases towards the edge. The sensitive area of the heat flux sensor is generally small, and responds to the peak of the distribution. However, the aperture size of the transfer standard cavity-type electrical substitution radiometer is much larger. Hence, the response of the radiometer will be proportional to the average flux captured from the distribution. This introduces a correction to the radiometer measurement to determine the peak value of the distribution from the average reading. This correction is estimated by considering the variation of the configuration factor across the radiometer aperture [5]. For the calibration conditions in the 51 mm VTBB, this correction is less than 0.1 %.
- Radiometer/Sensor reading: The sensor and the radiometer readings are averaged over a period of 10 s to 60 s depending on the heat flux level to account for the variance in the measurements due to purge gas flow effects. The purge gas effect is higher at lower heat flux levels (< 10 kW/m^2^) when the sensor is close to the blackbody exit, stations A and B for short and long extensions, respectively. The effect diminishes rapidly when the sensor is moved away from the exit. The maximum value of the standard deviation of the mean was about 0.3 % of the mean when the sensor was close to the blackbody exit.
- Repeat tests: The transfer calibration was performed at three different locations using both short and long extensions installed on the blackbody. Similar calibrations have been performed in the past in the 25 mm VTBB. The standard deviation of the responsivity from these calibrations is about 0.6 % of the mean value. This value of uncertainty is conservatively added to other uncertainty components to account indirectly for purge gas and other effects, which are otherwise difficult to evaluate.
- Combined uncertainty: The individual uncertainties have been listed in Table 3 and the combined uncertainty (*u*_c_) determined by calculating the square root of the sum-of-the-squares of individual uncertainties. The relative expanded uncertainty (*U*) is about ±2 % for a coverage factor of *k* = 2.

## 6. Conclusion

A new 51 mm diameter graphite tube blackbody facility developed for calibration of heat flux sensors is described. The graphite tube blackbody, heated electrically, operates up to a maximum temperature of 2773 K. A closed-loop cooling system using a water-to-water heat exchanger cools electrodes and the outer reflecting shield. The facility developed was successfully used to calibrate a Schmidt-Boelter heat flux sensor using the transfer technique with reference to an electrical substitution radiometer. The transfer calibration results agreed with the previous data within the experimental uncertainty limits.

## Figures and Tables

**Table 1:** Location of measurement stations with reference to blackbody aperture

	Station-A		Station-B		Station-C	
	*d*, *d*_1_	*θ, θ*_1_	*d*, *d*_1_	*θ, θ*_1_	*d*, *d*_1_	*θ, θ*_1_
Short extension						

Distance from exit (*d*_1_)	2.54 cm	45.0°	10.2 cm	14.0°	25.4 cm	5.7°
Distance from aperture (*d*)	20.0 cm	7.3°	27.6 cm	5.3°	42.8 cm	3.4°

Long extension						

Distance from exit (*d*_1_)			2.54 cm	45.0°	17.8 cm	8.1°
Distance from aperture (*d*)			27.9 cm	5.2°	43.1 cm	3.4°

**Table 2:** Measured responsivity of reference standard Schmidt-Boelter gage at different locations from the blackbody exit in the 51 mm VTBB

51 mm VTBB (short extension)									
Station	A			B			C		
	*d* (cm)		*d/a*	*d* (cm)		*d/a*	*d* (cm)		*d/a*
Distance from aperture	20.0		3.94	27.6		5.43	42.8		8.43
Responsivity mV/(kW/m^2^)		0.121			0.120			0.119	

Average	0.120 mV/(kW/m^2^)								

51 mm VTBB (long extension)									
Station	A			B			C		
	*d* (cm)		*d/a*	*d* (cm)		*d/a*	*d* (cm)		*d/a*

Distance from aperture				27.9		5.49	43.1		8.48
Responsivity mV/(kW/m^2^)					0.120			0.120	

Average	0.120 mV/(kW/m^2^)								

25 mm VTBB measurements	0.119 mV/(kW/m^2^)								

*d*: distance between aperture and sensor, *a*: blackbody aperture diameter.

**Table 3:** Estimate of uncertainties in heat flux sensor calibration(Heat flux range 10 kW/m^2^ to 50 kW/m^2^)

Uncertainty Source		Type	Uncertainty [%]
a. Transfer standard ESR		B	0.60
b. Blackbody:	Temperature	B	0.04
	Emissivity	B	0.00
	Aperture uniformity	B	0.00
c. Alignment:	Linear (Station A)	B	0.20
	Angular	B	0.06
d. Radiometer aperture averaging effect		B	0.10
e. Output reading:	Radiometer	A	0.30
	Heat flux sensor	A	0.30
f. Repeat tests on a similar gage		A	0.60
Relative expanded uncertainty (*U* = *k* · u_c_)		k = 2	±2.1

## Footnotes

1 Used to define an interval about the measurement result within which the value of the measurand is confidently believed to lie. A coverage factor of 2 represents a confidence level of 95.45 % for an ideal Gaussian uncertainty distribution.

2 Manufactured by Thermo Gauge Instrument Inc., P. O. Box 1457, Fort Ashby, WV 26719.

3 Certain commercial equipment, instruments, or materials are identified in this paper to foster understanding. Such identification does not imply recommendation or endorsement by the National Institute of Standards and Technology, nor does it imply that the materials or equipment are necessarily the best available for the purpose.
